# Periodontitis Primes the Oral Microenvironment for PS-Dependent Non-Canonical Entry Pathways Linked to SARS-CoV-2 Susceptibility

**DOI:** 10.21203/rs.3.rs-9163482/v1

**Published:** 2026-04-13

**Authors:** Araceli Valverde, Kristelle Capistrano, Raza Ali Naqvi, Sarah Elshourbagy, Sodabeh Etminan, Gloria Sandoval, Maria Bambrilla, Salvador Nares, Deepak Shukla, Joel Schwartz, Afsar Naqvi

**Affiliations:** 1 Department of Periodontics, University of Illinois Chicago, Chicago, Illinois, USA, 60612; 2 Mile Square Health System, University of Illinois Chicago, Chicago, Illinois, USA, 60608; 3 Posgrado de Periodoncia, Facultad de Odontología, Universidad Autónoma de Nuevo León, Monterrey, México, 64460; 4 Department of Oral Medicine and Diagnostic Sciences, University of Illinois Chicago, Chicago, Illinois, USA, 60612; 5 Department of Ophthalmology and Visual Sciences, University of Illinois Chicago, Chicago, Illinois, USA, 60612; 6 Department of Microbiology and Immunology, University of Illinois Chicago, Chicago, Illinois, USA, 60612

**Keywords:** SARS-CoV-2, Oral Mucosal Immunity, Oral Biology, Long Covid, Phospholipids phosphatidylserine (PS)

## Abstract

SARS-CoV-2 infection extends beyond the respiratory tract, with the oral cavity emerging as a critical site of viral activity shaped by epithelial receptor expression, microbial interactions, and inflammatory status. Periodontal disease (PD), a chronic dysbiotic condition, may heighten susceptibility to SARS-CoV-2 through inflammation-driven upregulation of non-canonical viral entry pathways. In this study, we investigated genes in the phosphatidylserine (PS)–dependent pathway, including ADAM17, ATP11c, TIM1, TIM3, and TIM4, in gingival tissue, saliva, and oral keratinocytes to define how PD and SARS-CoV-2 coordinately modulate oral viral entry mechanisms. Prepandemic gingival biopsies revealed significant upregulation of all non-canonical receptors in inflamed tissue, indicating that PD alone establishes a permissive molecular environment for PS-mediated microbial entry. In a post-vaccination cohort, salivary expression of these receptors was markedly elevated in COVID-19–positive individuals with PD, accompanied by significantly increased salivary PS levels, suggesting synergistic effects of viral exposure and periodontal inflammation. *In vitro* co-infection of primary human oral keratinocytes with SARS-CoV-2 and periodontal pathogens (*P. gingivalis*, *A. actinomycetemcomitans*) induced robust, synergistic activation of PS-dependent entry genes, particularly TIM family receptors. Together, these findings identify PS and its associated receptors as inflammation-responsive mediators that expand SARS-CoV-2 entry routes in the oral mucosa. This work highlights a mechanistic intersection between COVID-19 and periodontal disease, with implications for viral persistence, immune dysregulation, and long-term oral health outcomes.

## Introduction

The coronavirus disease 2019 (COVID-19), caused by severe acute respiratory syndrome coronavirus 2 (SARS-CoV-2), has profoundly altered global health landscapes. While the respiratory tract has been the primary focus of investigation, emerging evidence underscores the oral cavity as a critical site of viral activity [1–3]. The oral mucosa expresses angiotensin-converting enzyme 2 (ACE2) and transmembrane serine proteases (TMPRSS), providing a portal of entry and a reservoir for viral persistence [4,7]. This environment not only facilitates viral replication but also enables immune evasion, thereby contributing to both acute infection and the development of post-acute sequelae conditions (PASC) [5]. Oral manifestations such as dysgeusia, xerostomia, and gingival inflammation highlight the importance of oral health in the broader context of COVID-19 pathogenesis [2,6].

The canonical pathways of SARS-CoV-2 entry involve receptor-mediated interactions with ACE2 and members of the TMPRSS family, including TMPRSS2, TMPRSS4, and TMPRSS11D [4,7,8]. These entry factors are differentially expressed across oral tissues, with the salivary glands and lingual epithelium showing higher expression than palatal or buccal sites [1,7]. Nevertheless, knowledge of the precise mechanisms governing SARS-CoV-2 entry and persistence in the oral cavity remains limited. Emerging evidence suggests that noncanonical pathways may also contribute to viral entry. Microbially derived metabolites and proteolytic enzymes can mimic host proteases such as furin, promoting cleavage of the viral spike protein and exposing motifs that enhance membrane fusion [8–10]. In this context, oral microbial activity may facilitate SARS-CoV-2 infection through mechanisms that partially bypass canonical receptor-dependent entry [9,10]. Together, these findings broaden the mechanistic understanding of SARS-CoV-2 infection and highlight the complex interplay among viral tropism, host receptor expression, and the oral microbiome [10].

Periodontal disease (PD), a chronic inflammatory condition driven by oral microbiome dysbiosis, has emerged as a significant comorbidity influencing susceptibility to SARS-CoV-2 infection [11–13]. PD-associated pathogens release metabolites and proteases that overlap with viral entry pathways, thereby facilitating SARS-CoV-2 adherence and persistence [9,12]. Dysbiosis-induced inflammation, characterized by elevated cytokines such as interleukin-6 (IL-6) and tumor necrosis factor-alpha (TNFα), skews immune regulation and diminishes viral clearance [12,14]. Consequently, individuals with pre-existing PD may experience exacerbated oral and systemic complications following COVID-19 infection [11–13]. This bidirectional relationship between poor oral health and viral pathogenesis highlights the importance of maintaining oral immunity as a determinant of infection outcomes.

Persistent SARS-CoV-2 in the oral mucosa may contribute to post-acute sequelae of SARS-CoV-2 infection (PASC) by promoting chronic low-level infection and immune exhaustion [5,15]. Noncanonical mechanisms, including microbial enzyme-mediated entry and dysbiosis-driven immune suppression, may further sustain infection and worsen periodontal disease after COVID-19 [9,11,12]. In addition to the ACE2/TMPRSS pathway, alternative host factors such as ADAM17, ATP11C, TIM1, TIM3, and TIM4 may facilitate viral entry and persistence [16–18]. Together, these mechanisms may create a feed-forward cycle of inflammation, impaired viral clearance, and periodontal tissue damage. Understanding both canonical and noncanonical pathways is important for clarifying how COVID-19 exacerbates PD and for identifying targeted interventions to reduce long-term oral and systemic sequelae.

Phosphatidylserine (PS) is an important molecular signal that becomes exposed on the outer leaflet of host cell membranes during apoptosis, cellular stress, and inflammation, and many pathogens exploit this change to gain entry [17]. Enveloped viruses such as Ebola, Dengue, Zika, Vaccinia, and SARS-CoV-2 use PS-mediated apoptotic mimicry to engage TIM receptors, facilitating attachment, internalization, and immune evasion [19–21]. Some bacteria also take advantage of PS exposure or PS-binding receptors to enhance adherence and uptake [22]. This mechanism may be especially important in inflamed tissues, where cytokine-induced stress promotes PS externalization and increases the expression of receptors such as ADAM17, ATP11C, TIM1, TIM3, and TIM4 [16–18].

Here, we show that periodontal inflammation alone elevates noncanonical receptor expression in gingival tissue and saliva, while SARS-CoV-2 infection further enhances this response and is accompanied by substantial increases in salivary PS. Co-infection studies reinforced this relationship, demonstrating that viral–bacterial interactions strongly induce PS-pathway genes in gingival keratinocytes. Collectively, these results suggest that PS and its receptors are inflammation-responsive mediators that may expand SARS-CoV-2 entry routes, worsen immune dysregulation, and deepen the interplay between COVID-19 and periodontal disease.

## Material and Methods

### Ethical Approval

This study was conducted in accordance with the Declaration of Helsinki and approved by the Ethics Committee of the Facultad de Odontología, Universidad Autónoma de Nuevo León, Monterrey, Mexico, as well as the Institutional Review Board of the University of Illinois Chicago College of Dentistry (IRB #2015–1093; IRB #2016–0696). Written informed consent was obtained from all participants, and all procedures complied with HIPAA requirements to ensure confidentiality.

### Study Populations

#### Periodontal disease cohort:

Subjects aged 18 to 65 years who were in good systemic health were recruited from the Postgraduate Periodontics Clinic at the Universidad Autónoma de Nuevo León. Healthy controls (n = 8) were defined as individuals with probing depths of ≤3 mm, no bleeding on probing, and no radiographic evidence of bone loss. Patients with periodontitis (n = 8) had probing depths of ≥6 mm, bleeding on probing, and radiographic evidence of alveolar bone loss. Individuals with chronic systemic diseases, including diabetes, hepatitis, renal failure, clotting disorders, or HIV infection, were excluded, as were those who had recently received antibiotic therapy or were taking medications known to affect periodontal status, such as phenytoin, calcium channel blockers, or cyclosporine.

#### COVID-19 cohort:

Adults aged 21 to 70 years were recruited through the Miles Square Health Center and the University of Illinois Chicago College of Dentistry. Individuals with active lung disease or current COVID-19 infection were excluded.

### Sample Collection

#### Gingival tissue:

Gingival biopsies were obtained from periodontally healthy subjects during crown-lengthening procedures. Samples included gingival epithelium, col tissue, and underlying connective tissue, and were harvested using intrasulcular and inverse bevel incisions approximately 2 mm from the free gingival margin. Tissues were immediately placed in RNAlater (Qiagen) and stored at −80°C.

#### Saliva:

Participants refrained from eating, rinsing, or toothbrushing for at least 1 hour before sample collection. Saliva was collected by having subjects chew sterile paraffin wax for 5 minutes and expectorate into sterile 50 mL conical tubes. A salivary flow rate of <5 mL per 5 minutes was considered indicative of xerostomia. Saliva viscosity was categorized as either mucoid/stringy or watery. Aliquots were prepared for flow cytometry and cytologic analysis. Samples designated for RNA analysis were stabilized in QIAzol (Qiagen) and stored at −80°C, while DNA was isolated using Qiagen kits according to the manufacturer’s instructions.

### Cell Culture

Primary human oral keratinocytes (HOKs) were obtained from ScienCell Research Laboratories (Carlsbad, CA, USA). Cells were authenticated by immunofluorescence for oral keratinocyte markers, including cytokeratins 8, 18, and 19, and tested negative for HIV-1, HBV, HCV, fungi, bacteria, and mycoplasma. HOKs were maintained in DermaLife K Keratinocyte Medium Kit (Lifeline Cell Technology, Frederick, MD, USA) supplemented with 10% fetal bovine serum, 100 U/mL penicillin, 100 μg/mL streptomycin, and 25 ng/mL amphotericin under standard culture conditions (37°C, 5% CO_2_).

### SARS-CoV-2 and Periodontal Bacterial Challenge in Oral Keratinocytes

HOKs were coinfected with SARS-CoV-2 strain Omicron (0.5 MOI) and supernatant from periodontal pathogens (*P. gingivalis* and *A. actinomycetemcomitans* [both 1:10]). After treatment, cells were washed three times with PBS, lysed in TRIzol reagent (Invitrogen), and processed for RNA isolation.

### Total RNA Isolation, cDNA Synthesis, and Quantitative PCR

Total RNA isolation and downstream analyses were performed uniformly across gingival tissue, saliva, and cell culture samples. RNA was extracted using the miRNeasy Micro Kit (Qiagen, Gaithersburg, MD, USA). cDNA was synthesized from 250 ng of total RNA using the High-Capacity cDNA Reverse Transcription Kit (Applied Biosystems, USA). Gene expression was analyzed by RT-qPCR using SYBR Green Gene Expression Master Mix (Applied Biosystems, USA) on a StepOne 7500 thermocycler (Applied Biosystems, USA). Expression levels of ATP11C, ADAM17, TIM1, TIM3, TIM4, RgpA (Pg), Rpl2 (Aa), and BspA (Tf), together with the housekeeping gene β-actin (Millipore Sigma), were measured using primers listed in Table 1. Relative expression was calculated using the 2^−ΔΔCt^ method based on triplicate Ct values.

### Phosphatidylserine ELISA

Phosphatidylserine (PS) levels in saliva were measured using a PS ELISA Kit (AFG Scientific) in COVID-19-positive individuals with periodontitis (n = 10) and COVID-19-negative individuals with periodontitis (n = 10), with both groups compared to periodontally healthy controls (n = 6). Saliva samples were centrifuged at 1000 × g for 15 minutes at 2–8°C, and the supernatants were collected, aliquoted, and stored at −80°C until analysis. PS concentrations were quantified using a competitive inhibition ELISA. Briefly, microtiter wells pre-coated with PS were incubated with 50 μL of standards or saliva samples together with 50 μL of biotin-conjugated anti-PS antibody. After incubation for 1 hour at 37°C, plates were washed and incubated with streptavidin-HRP for 60 minutes at 37°C. Following five washes, 90 μL of TMB substrate was added and incubated for 20 minutes in the dark. The reaction was terminated with 50 μL of stop reagent, and absorbance was measured at 450 nm. PS concentrations were determined by interpolation from a standard curve generated by linear regression and corrected for sample dilution.

### Statistical Analysis

Data were analyzed using GraphPad Prism (La Jolla, CA, USA). Results are presented as mean ± SD or mean ± SEM from three independent replicates, and all experiments were performed at least three times. Dataset normality was assessed using the Shapiro-Wilk test prior to statistical analysis. Comparisons between two groups were performed using the Student’s t-test, with p < 0.05 considered statistically significant. To evaluate associations among multiple study groups, one-way and two-way ANOVA were also performed, with p < 0.01 considered significant where applicable.

## Results

### 1. Non-Canonical Viral Entry Receptors Are Upregulated in Inflamed Human Gingiva Collected Pre-Pandemic

To determine whether periodontal inflammation contributes to noncanonical viral entry pathways, we examined gingival expression of multiple genes implicated in this process in a cohort collected before the COVID-19 pandemic. Periodontal inflammation is known to alter epithelial immune signaling and receptor expression [23–25], and several PS-associated receptors—including ADAM17, ATP11C, TIM1, TIM3, and TIM4—have been implicated in viral entry and apoptotic mimicry [16–21].

**Figure 1.**
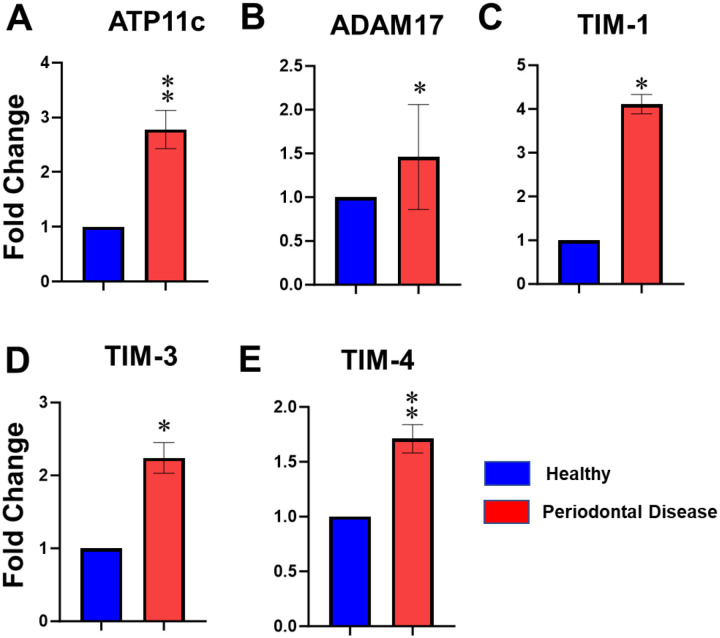
Periodontal inflammation is associated with robust upregulation of the non-canonical SARS-CoV-2 entry receptor pathway in human gingiva. Gingival biopsies were collected from patients with or without periodontal disease (n=10/group). Total RNA was isolated and the expression of genes involved in PS-dependent non-canonical SARS-CoV-2 entry pathway, including ADAM17, ATP11c, TIM1, TIM3, and TIM4, was quantified by RT-qPCR analysis. Each bar shows the mean ± SD. Student’s *t* tests were used to calculate *p* values, and *p* < 0.05 was considered significant. *p < 0.05, **p < 0.01, ***p < 0.001.

We performed ex vivo analyses of gingival biopsies from periodontally healthy and diseased individuals and quantified the expression of noncanonical pathway-associated genes, including ATP11C, ADAM17, TIM1, TIM3, and TIM4, by RT-qPCR (Figure 1A–E). Relative to healthy controls, all targets were significantly upregulated in diseased gingiva, including ATP11C (2.78-fold, p < 0.01), ADAM17 (1.46-fold, p < 0.05), TIM1 (4.11-fold, p < 0.05), TIM3 (2.24-fold, p = 0.0145), and TIM4 (1.71-fold, p < 0.01). Because these biopsies were collected before the COVID-19 pandemic, these findings indicate that periodontal disease alone can generate a molecular microenvironment enriched in PS-dependent, noncanonical entry receptors [16–21], potentially facilitating microbial entry and contributing to downstream immune dysregulation.

### 2. Salivary Expression of Noncanonical Entry Receptors Is Elevated in a Post-Vaccination Cohort of COVID-19 Patients with Gingival Inflammation

The convergence of PD and SARS-CoV-2 infection may create a distinct oral microenvironment in which altered salivary expression of noncanonical receptors reveals novel mechanisms of viral susceptibility and host–pathogen interaction. SARS-CoV-2 infects oral epithelial and salivary gland tissues [1,7,26], and periodontal inflammation amplifies receptor expression and protease activity relevant to viral entry [23–25,9].

**Figure 2.**
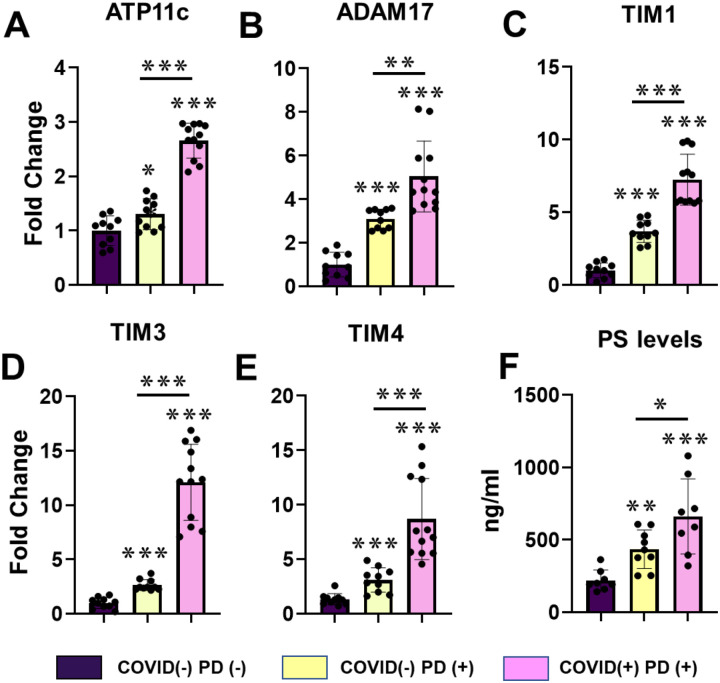
Salivary expression of non-canonical sars-cov-2 entry pathway genes and PS levels in post-vaccination covid-19 patients with gingival inflammation. **A)** Salivary expression of non-canonical SARS-CoV-2 entry pathway–associated genes (ATP11c, ADAM17, TIM1, TIM3, TIM4) was assessed in post-vaccination COVID-19–positive individuals with periodontal disease (COVID-19+/PD+, n = 12), COVID-19–negative individuals with periodontal disease (COVID-19−/PD+, n = 12), and periodontally healthy SARS-CoV-2–negative controls (n = 12). Each bar shows the mean ± SEM. Gene expression levels were quantified by qPCR and compared across clinical groups using ANOVA and Kruskal–Wallis tests with Dunn’s post hoc analysis. **B)** Salivary PS concentrations were measured by ELISA in post-vaccination COVID-19+/PD+ subjects (n = 10), COVID-19-/PD+ subjects (n = 10), and periodontally healthy controls (n = 6). Data are shown as mean ± SEM. Statistical comparisons were performed using ANOVA and the Kruskal-Wallis test with Dunn’s post hoc correction.

To investigate this possibility, we quantified salivary expression of noncanonical pathway-associated genes in COVID-19-positive individuals with periodontitis (n = 12) and COVID-19-negative individuals with periodontitis (n = 12), and compared both groups with periodontally healthy, SARS-CoV-2-negative controls (n = 12). Relative to controls, expression of all genes was significantly increased in both periodontitis groups, with the highest levels consistently observed in the COVID-19+/PD+ cohort (Figure 2A–E). Together, these findings demonstrate that salivary noncanonical receptor expression is elevated in PD and is further amplified in individuals with prior SARS-CoV-2 infection, consistent with known oral epithelial susceptibility to SARS-CoV-2 [1,7,26] and inflammation-driven receptor modulation [23–25,9].

### 3. Post-Vaccination COVID-19 Patients with Gingival Inflammation Exhibit Elevated Salivary PS Expression

Altered salivary PS expression emerges as a defining feature in COVID-19 patients with gingival inflammation, reflecting a perturbed oral mucosal milieu in which systemic viral immune disturbances intersect with local periodontal responses. PS externalization is a hallmark of cellular stress and inflammation [17], and PS-mediated apoptotic mimicry is exploited by multiple enveloped viruses—including SARS-CoV-2—to engage TIM receptors and enhance entry [19–21].

We analyzed salivary PS expression levels in post-vaccinated COVID-19–positive individuals with PD (n = 10), COVID-19–negative individuals with PD (n = 10), and periodontally healthy controls (n = 6) (Figure 2F). Salivary PS expression was significantly elevated in post-vaccination COVID-19 patients presenting with gingival inflammation. Group-wise comparisons confirmed a robust overall difference among groups (overall p < 0.0002), indicating that PS upregulation is a consistent feature of COVID-19–positive subjects and aligns with PS-dependent viral entry mechanisms [19–21].

### 4. Salivary Periodontal Bacterial Burden Is Elevated in COVID-19 with Comorbid Periodontitis

The oral cavity represents a key interface between periodontal inflammation and respiratory viral infection, making saliva a practical, noninvasive matrix to assess whether COVID-19 is associated with shifts in periodontal dysbiosis. Periodontal pathogens such as *A. actinomycetemcomitans*, *P. gingivalis*, and *T. forsythia* are well-established drivers of dysbiosis and inflammatory amplification [23–25,12], and microbial proteases can enhance SARS-CoV-2 spike activation [9,13].

**Figure 3.**
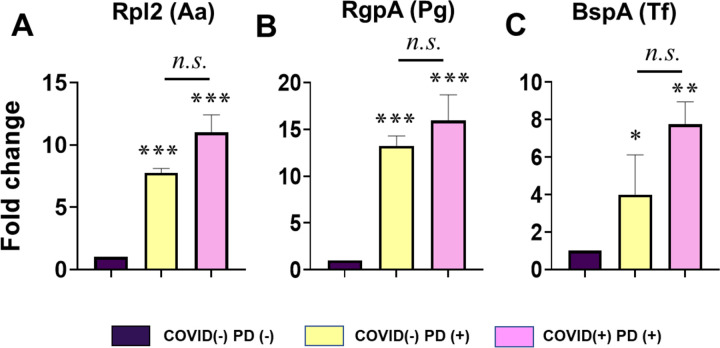
Elevated salivary periodontal pathogen burden in COVID-19 subjects with comorbid periodontitis. Saliva samples were collected from study participants and analyzed by RT-qPCR to quantify transcripts from three established periodontal pathogens: *Aggregatibacter actinomycetemcomitans* (Aa), *Porphyromonas gingivalis* (Pg), and *Tannerella forsythia* (Tf). Target genes included Aa ribosomal protein L2 (RPL2), Pg arginine-specific gingipain A (RgpA), and Tf BspA. Bar graphs show relative expression (fold-change) in COVID(−)/PD(+) and COVID(+)/PD(+) groups normalized to the control group. Quantitative RT-qPCR analysis is shown for (A) Aa-encoded RPL2, (B) Pg RgpA, and (C) Tf BspA. Data are presented as mean ± SEM from three independent experiments. Statistical significance was determined using Student’s t test, with p < 0.05 considered significant (*p < 0.05, **p < 0.01, ***p < 0.001).

In our preliminary analysis, the COVID-19+/PD+ cohort exhibited higher salivary levels of these pathogens relative to the other cohorts (Figure 3A–C). Overall, pathogen levels were highest in COVID-19+/PD+, suggesting that COVID-19 positivity in periodontitis is associated with a greater salivary burden of periodontopathic bacteria, consistent with cooperative effects between SARS-CoV-2 infection and periodontal dysbiosis [12–14].

### 5. Co-Infection with SARS-CoV-2 and Periodontal Bacteria Induces PS-Dependent Non-Canonical Entry Pathway Genes in Gingival Keratinocytes

Periodontal bacteria, including *P. gingivalis* and *A. actinomycetemcomitans*, drive inflammation in the gingiva [23–25,12], which is also a site for SARS-CoV-2 infection [1,7,26]. Viral–bacterial interactions are known to synergistically modulate host receptor expression and protease activity [9,13,14], and PS-dependent receptors such as TIM1, TIM3, TIM4, ADAM17, and ATP11C facilitate apoptotic mimicry-mediated viral entry [16–21].

**Figure 4.**
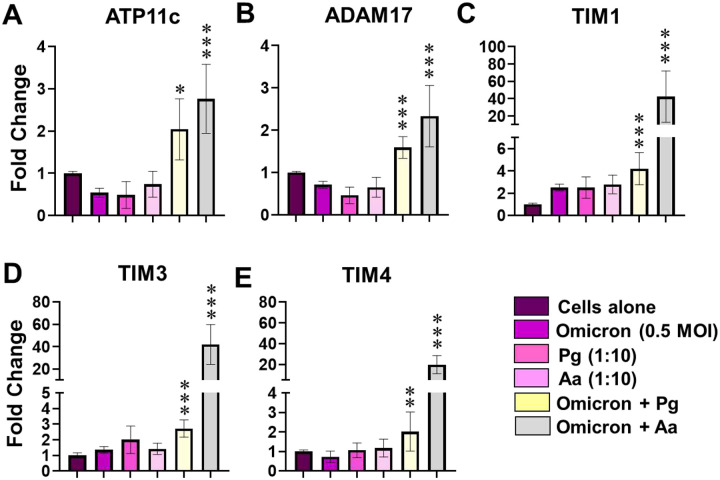
Viral-bacterial coinfection enhances expression of phosphatidylserine-dependent noncanonical entry receptors in oral keratinocytes. Primary human gingival keratinocytes were infected with SARS-CoV-2 Omicron (0.5 MOI), P. gingivalis (Pg), A. actinomycetemcomitans (Aa), or the indicated coinfection conditions (Omicron + Pg or Omicron + Aa) for 24 h. Transcript expression of phosphatidylserine (PS)-dependent noncanonical SARS-CoV-2 entry receptors, including ADAM17, ATP11C, TIM3, and TIM4, was measured by RT-qPCR. Data are presented as mean ± SD. Statistical significance was determined using Student’s t test, with p < 0.05 considered significant (*p < 0.05, **p < 0.01, ***p < 0.001).

Co-infection with SARS-CoV-2 and periodontal bacteria significantly increased expression of PS-pathway–associated receptors compared with untreated cells and relative to individual pathogens (Figure 4A–E). These results indicate that concurrent exposure to SARS-CoV-2 and periodontal bacteria drives a distinct transcriptional program in gingival keratinocytes characterized by robust induction of PS-dependent noncanonical entry receptors, consistent with known roles of PS-mediated viral entry [19–21] and inflammation-driven receptor modulation [16–18].

## Discussion

PD is a chronic inflammatory condition driven by oral microbial dysbiosis, and its effects extend well beyond local tissue destruction. The persistent imbalance in the periodontal microbiome alters host immune responses, sustains inflammatory signaling, and disrupts mucosal homeostasis [27–29]. Emerging evidence suggests that individuals with poor oral health may serve as reservoirs for pathogenic oral bacteria, thereby maintaining a dysbiotic environment that can favor viral persistence and worsen oral pathology [30]. Oral dysbiosis, a defining feature of periodontitis, may influence susceptibility to SARS-CoV-2, affect viral clearance, and contribute to the development of post-acute sequelae of SARS-CoV-2 infection (PASC) [31,32]. This work reveals a previously underappreciated intersection between periodontal inflammation and SARS-CoV-2 susceptibility, centered on the dysregulation of PS and its associated non-canonical viral entry receptors. PD establishes a chronically inflamed, dysbiotic environment that elevates PS exposure and primes gingival tissues for alternative viral entry routes [33,34]. The synergistic induction of these receptors during viral–bacterial coinfection underscores the oral cavity as a dynamic interface where dysbiosis, inflammation, and viral exploitation converge [35].

Our findings show that periodontal inflammation alone is sufficient to upregulate a panel of phosphatidylserine (PS)-dependent, noncanonical viral entry receptors in gingival tissue, even in the absence of SARS-CoV-2 exposure. In pre-pandemic gingival biopsies, ADAM17, ATP11C, TIM1, TIM3, and TIM4 were all significantly elevated in diseased tissue, indicating that PD establishes a molecular environment permissive to PS-mediated microbial entry. This baseline increase suggests that individuals with periodontitis may be predisposed to alternative entry mechanisms that operate alongside, or independently of, canonical ACE2/TMPRSS pathways [36,37], potentially increasing vulnerability to SARS-CoV-2 and other enveloped viruses that use apoptotic mimicry [38].

The post-vaccination clinical cohort further highlights the synergistic relationship between PD and SARS-CoV-2 exposure. Salivary expression of noncanonical receptors was markedly higher in COVID-19-positive individuals with periodontitis than in those with periodontitis alone. This amplification was accompanied by significantly elevated salivary PS levels, suggesting that SARS-CoV-2 infection intensifies inflammatory and membrane-remodeling processes that are already active in periodontal tissues. Because PS externalization is a hallmark of cellular stress and inflammation [39], its increased abundance in saliva may reflect enhanced epithelial turnover, immune activation, or viral-bacterial interactions within the oral cavity [30,35]. Together, these findings support a model in which SARS-CoV-2 and PD converge to strengthen PS-dependent entry pathways, thereby broadening the range of potential viral access routes in the oral mucosa.

Our in vitro coinfection experiments provide mechanistic support for this model. Gingival keratinocytes exposed simultaneously to SARS-CoV-2 and periodontal pathogens showed robust induction of PS-dependent receptors, particularly members of the TIM family. This finding is consistent with prior work demonstrating that enveloped viruses can exploit PS receptors, including TIM family proteins, through apoptotic mimicry to enhance host-cell attachment and uptake [38,40–42]. The pronounced upregulation observed during coinfection with *A. actinomycetemcomitans* suggests that specific periodontal pathogens may disproportionately amplify these noncanonical entry programs. Although direct evidence that periodontal pathogens themselves use PS-receptor-mediated entry remains limited, substantial literature shows that major periodontal pathogens disrupt epithelial homeostasis, trigger inflammatory signaling, alter cell-death pathways, and interfere with apoptotic cell clearance [27,28,44], thereby creating tissue conditions in which PS exposure and PS-responsive receptor systems are likely to be enhanced. Thus, microbial proteases, inflammatory mediators, and membrane-remodeling processes associated with dysbiosis may increase the availability of host factors that facilitate viral fusion, uptake, or persistence [35,44].

The implications of these observations extend beyond viral entry and point to broader effects on immune regulation and long-term disease outcomes. PD is characterized by chronic elevations in IL-6, TNF-α, and other proinflammatory mediators [29,31], in part through disrupted immunoregulatory pathways such as altered Treg responses. This inflammatory milieu can impair mucosal defense and reduce the efficiency with which viral and bacterial pathogens are cleared. Upregulation of ADAM17, a sheddase involved in the release of multiple immunoregulatory and inflammatory mediators, may further contribute to epithelial dysfunction and immune imbalance [36]. Likewise, TIM family receptors, which recognize PS on apoptotic cells and viral envelopes, may support immune evasion by promoting tolerogenic or suppressive signaling [38,40]. Together, these processes suggest a feed-forward loop in which dysbiosis, inflammation, and impaired immune regulation reinforce one another, potentially enabling persistent SARS-CoV-2 activity in the oral cavity and contributing to PASC-associated manifestations such as dysgeusia, xerostomia, and chronic mucosal inflammation [2,5,6].

Collectively, our data identify PS and its associated receptors as inflammation-responsive mediators linking PD with SARS-CoV-2 susceptibility. Periodontitis appears to establish a pre-existing molecular landscape enriched for noncanonical entry pathways, while SARS-CoV-2 infection further amplifies these responses in the context of ongoing dysbiosis. This interaction may help explain why individuals with poor oral health experience more severe or prolonged COVID-19 outcomes [11–13] and underscores the oral cavity as an important site of viral-bacterial synergy. These findings also suggest new therapeutic opportunities, including strategies aimed at reducing oral inflammation, limiting PS exposure, or modulating TIM family signaling [38–40]. Periodontal health may represent a modifiable factor that could reduce susceptibility to SARS-CoV-2, lessen long-term sequelae, and improve mucosal immune resilience.

## Conclusion

By identifying PS-dependent mechanisms as a shared molecular axis linking PD and SARS-CoV-2, our findings provide a mechanistic framework that may help explain prolonged viral activity, impaired mucosal immunity, and the oral manifestations observed in post-acute sequelae of SARS-CoV-2. These insights highlight the importance of oral health as a modifiable factor influencing susceptibility to respiratory viruses and suggest that interventions aimed at reducing inflammation, restoring microbial balance, or targeting PS-mediated entry pathways may hold therapeutic potential. Strengthening periodontal health may therefore represent a practical and impactful strategy to mitigate viral persistence, improve mucosal immune resilience, and reduce the burden of long-term COVID-19–related complications.

## Figures and Tables

**Table 1:** Primer sets used for quantitative RT-PCR analysis of various human and bacterial genes.

Gene	Forward Primer (5’ → 3’)	Reverse Primer (5’ → 3’)
ATP11c	CTTCCTTGTACAGGTCACAGT	GTCCCAAAGACCTGGCAACA
ADAM17	AACAGCGACTGCACGTTGAAGG	CTGTGCAGTAGGACACGCCTTT
TIM1	CTTCAGCTACCCAGTGACACTG	TCTCCGTCTAGTGGTGTCTCTGG
TIM3	GACTCTAGCAGACAGTGGGATC	GGTGGTAAGCATCCTTGGAAAGG
TIM4	AAGCCACAGGTCTTCTGACTCC	AGCAGGACAGTGTCAGCAGAAG
β-Actin	CACCATTGGCAATGAGCGGTTC	AGGTCTTTGCGGATGTCCACGT
Bacterial gene transcripts: *P. gingivalis* (Pg), *A. actinomycetemcomitans* (Aa), and *T. forsythia* (Tf)		
Pg RgpA gingipain	AGTTCAATCCTGTAAAGAAC	TCTGCTGCGAGCACAACCTT
Aa Ribosomal L2	TACAGATCATTGCCCGTGAA	GCTTTACCCAATACGCGAAG
Tf BspA	GAAGCGAAGGACGTATGGA	TTGGTGATGGTGAGGGTTTTG

## Data Availability

The datasets generated and/or analyzed during the current study are available from the corresponding author on reasonable request.
